# Comprehensive analysis of human microRNA target networks

**DOI:** 10.1186/1756-0381-4-17

**Published:** 2011-06-17

**Authors:** Jun-ichi Satoh, Hiroko Tabunoki

**Affiliations:** 1 Department of Bioinformatics and Molecular Neuropathology, Meiji Pharmaceutical University, 2-522-1 Noshio, Kiyose, Tokyo 204-8588, Japan

## Abstract

**Background:**

MicroRNAs (miRNAs) mediate posttranscriptional regulation of protein-coding genes by binding to the 3' untranslated region of target mRNAs, leading to translational inhibition, mRNA destabilization or degradation, depending on the degree of sequence complementarity. In general, a single miRNA concurrently downregulates hundreds of target mRNAs. Thus, miRNAs play a key role in fine-tuning of diverse cellular functions, such as development, differentiation, proliferation, apoptosis and metabolism. However, it remains to be fully elucidated whether a set of miRNA target genes regulated by an individual miRNA in the whole human microRNAome generally constitute the biological network of functionally-associated molecules or simply reflect a random set of functionally-independent genes.

**Methods:**

The complete set of human miRNAs was downloaded from miRBase Release 16. We explored target genes of individual miRNA by using the Diana-microT 3.0 target prediction program, and selected the genes with the miTG score ≧ 20 as the set of highly reliable targets. Then, Entrez Gene IDs of miRNA target genes were uploaded onto KeyMolnet, a tool for analyzing molecular interactions on the comprehensive knowledgebase by the neighboring network-search algorithm. The generated network, compared side by side with human canonical networks of the KeyMolnet library, composed of 430 pathways, 885 diseases, and 208 pathological events, enabled us to identify the canonical network with the most significant relevance to the extracted network.

**Results:**

Among 1,223 human miRNAs examined, Diana-microT 3.0 predicted reliable targets from 273 miRNAs. Among them, KeyMolnet successfully extracted molecular networks from 232 miRNAs. The most relevant pathway is transcriptional regulation by transcription factors RB/E2F, the disease is adult T cell lymphoma/leukemia, and the pathological event is cancer.

**Conclusion:**

The predicted targets derived from approximately 20% of all human miRNAs constructed biologically meaningful molecular networks, supporting the view that a set of miRNA targets regulated by a single miRNA generally constitute the biological network of functionally-associated molecules in human cells.

## Introduction

MicroRNAs (miRNAs) are a class of endogenous small noncoding RNAs conserved through the evolution. They mediate posttranscriptional regulation of protein-coding genes by binding to the 3' untranslated region (3'UTR) of target mRNAs, leading to translational inhibition, mRNA destabilization or degradation, depending on the degree of sequence complementarity [1]. During the biogenesis of miRNAs, the primary miRNAs (pri-miRNAs) are transcribed from the intra- and inter-genetic regions of the genome by RNA polymerase II, followed by processing by the RNase III enzyme Drosha into pre-miRNAs. After nuclear export, they are cleaved by the RNase III enzyme Dicer into mature miRNAs consisting of approximately 22 nucleotides. Finally, a single-stranded miRNA is loaded onto the RNA-induced silencing complex (RISC), where the seed sequence located at positions 2 to 8 from the 5' end of the miRNA plays a pivotal role in recognition of the target mRNA [2]. At present, more than one thousand of human miRNAs are registered in miRBase Release 16 http://www.mirbase.org. The 3'UTR of a single mRNA is often targeted by several different miRNAs, while a single miRNA concurrently reduces the production of hundreds of target proteins [3]. Consequently, the whole miRNA system (microRNAome) regulate greater than 60% of all protein-coding genes in a human cell [4]. By targeting multiple transcripts and affecting expression of numerous proteins, miRNAs play a key role in fine-tuning of diverse cellular functions, such as development, differentiation, proliferation, apoptosis and metabolism. Therefore, aberrant regulation of miRNA expression is deeply involved in pathological events that mediate cancers [5] and neurodegenerative disorders [6].

Recent advances in systems biology have made major breakthroughs by illustrating the cell-wide map of complex molecular interactions with the aid of the literature-based knowledgebase of molecular pathways [7]. The logically arranged molecular networks construct the whole system characterized by robustness, which maintains the proper function of the system in the face of genetic and environmental perturbations [8]. In the scale-free molecular network, targeted disruption of limited numbers of critical components designated hubs, on which the biologically important molecular interactions concentrate, efficiently disturbs the whole cellular function by destabilizing the network [9]. Therefore, the identification of the hub in the molecular network constructed by target genes of a particular miRNA helps us to understand biological and pathological roles of individual miRNAs. Recently, Hsu et al. studied the human microRNA-regulated protein-protein interaction (PPI) network by utilizing the Human Protein Reference Database (HPRD) and the miRNA target prediction program TargetScan [10]. They found that an individual miRNA often targets the hub gene of the PPI network, although they did not attempt to characterize relevant pathways, diseases, and pathological events regulated by miRNA target genes.

At present, the question remains to be fully elucidated whether a set of miRNA target genes regulated by an individual miRNA in the whole human microRNAome generally constitute the biological network of functionally-associated molecules or simply reflect a random set of functionally-independent genes. To address this question, we attempted to characterize molecular networks of target genes of all human miRNAs by using KeyMolnet, a bioinformatics tool for analyzing molecular interactions on the comprehensive knowledgebase.

## Materials and methods

### MicroRNA Target Prediction

The complete list of 1,223 human miRNAs was downloaded from miRBase Release 16 http://www.mirbase.org. We searched the target genes of individual miRNA on the Diana-microT 3.0 target prediction program (diana.cslab.ece.ntua.gr/microT), which was selected because of the highest ratio of correctly predicted targets over other prediction tools [11]. Diana-microT 3.0 calculates the miRNA-targeted gene (miTG) score that reflects the weighted sum of the scores of all conserved and non-conserved miRNA recognition elements (MRE) on the 3'UTR of the target mRNA. The miTG score correlates well with fold changes in suppression of protein expression [11]. To optimize the parameter of miRNA-target interaction, we considered the target genes with a cutoff of the miTG score equal to or larger than 20 as the highly reliable targets, because we found that the targets with the miTG score < 20 exhibited the significantly lower precision score, an indicator of correctness in predicted interactions [11], compared with those having the score ≧ 20 (p = 2.78E-08 by Mann-Whitney's U-test).

### Molecular Network Analysis

Ensembl Gene IDs of target genes retrieved by Diana-microT 3.0 were converted into the corresponding Entrez Gene IDs by using the DAVID Bioinformatics Resources 6.7 program http://david.abcc.ncifcrf.gov[12], where non-annotated IDs were deleted. Then, Entrez Gene IDs of miRNA target genes were uploaded onto KeyMolnet.

KeyMolnet is a tool for analyzing molecular interactions on the literature-based knowledgebase that contains the contents on 123,000 molecular relationships among human genes and proteins, small molecules, diseases, pathways and drugs, established by the Institute of Medicinal Molecular Design (IMMD) (Tokyo, Japan) [13-15]. The core contents are collected from selected review articles and textbooks with the highest reliability, regularly updated and carefully curated by a team of expert biologists. KeyMolnet contains a panel of human canonical networks constructed by core contents in the KeyMolnet library. They represent the gold standard of the networks, composed of 430 pathways, 885 diseases, and 208 pathological events. Detailed information on all the contents is available from IMMD http://www.immd.co.jp/en/keymolnet/index.html upon request.

We utilized the neighboring network-search algorithm that selects the set of miRNA target genes as starting points to generate the network around starting points within one path, composed of all kinds of molecular interactions, including direct activation/inactivation, transcriptional activation/repression, and the complex formation. By uploading the list of Entrez Gene IDs onto KeyMolnet, it automatically provides corresponding molecules and a minimum set of intervening molecules as a node on networks. The generated network was compared side by side with human canonical networks described above. The algorithm that counts the number of overlapping molecules and/or molecular relations between the extracted network and the canonical network identifies the canonical network showing the most statistically significant contribution to the extracted network. This algorithm is essentially based on that of the GO::TermFinder [16]. The significance in the similarity between the extracted network and the canonical network is scored following the formula, where O = the number of overlapping molecules and molecular relations for the pathway or overlapping molecules alone for the disease and the pathological event between the extracted network and the canonical network, V = the number of molecules and/or molecular relations located in the extracted network, C = the number of molecules and/or molecular relations located in the canonical network, T = the number of total molecules and/or molecular relations of KeyMolnet, currently composed of approximately 15,700 molecules and 123,000 molecular relations, and the × = the sigma variable that defines coincidence.(1)

## Results

### Molecular Network of MicroRNA Target Genes

**Figure 1:**
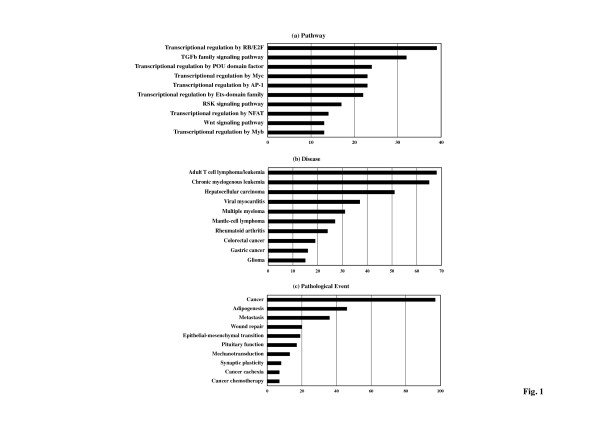
**The pathways, diseases, and pathological events relevant to 232 miRNA target networks**. Among 1,223 human miRNAs examined, Diana-microT 3.0 identified the set of reliable targets from 273 miRNAs. Among them, KeyMolnet extracted molecular networks from 232 miRNAs. The generated network was compared side by side with human canonical networks of the KeyMolnet library, composed of 430 pathways, 885 diseases, and 208 pathological events to identify the canonical network showing the most statistically significant contribution to the extracted network (see Table S1 for all the information). After top three pathways, diseases, and pathological events were individually totalized, the cumulated numbers of top 10 of **(a) **pathway, **(b) **disease, and **(c) **pathological event categories are expressed as a bar graph.

Among 1,223 human miRNAs examined, Diana-microT 3.0 predicted the targets from 532 miRNAs (43.5%). Among the 532 miRNAs, 273 miRNAs contained a set of highly reliable targets showing the miTG score ≧ 20. Among 273 miRNAs having reliable targets, KeyMolnet successfully extracted molecular networks from 232 miRNAs. They are comprised of 19% of total human miRNAs (microRNAome). Then, the generated network was compared side by side with human canonical networks of the KeyMolnet library, composed of 430 pathways, 885 diseases, and 208 pathological events. We found that not all 232 miRNAs contained entire categories of canonical networks because several miRNAs comprised relatively small numbers of targets. See Additional file 1 for all the information on 232 miRNAs and their target networks. When top three pathways, diseases, and pathological events were individually totalized, the most relevant pathway is 'transcriptional regulation by RB/E2F' (n = 39; 6.8% of total), followed by 'TGF-beta family signaling pathway' (n = 32; 5.6%) and 'transcriptional regulation by POU domain factor' (n = 24; 4.2%), the most relevant disease is 'adult T cell lymphoma/leukemia' (n = 68; 12.1%), followed by 'chronic myelogenous leukemia' (n = 65; 11.5%) and 'hepatocellular carcinoma' (n = 51; 9.1%), and the most relevant pathological event is 'cancer' (n = 97; 24.7%), followed by 'adipogenesis' (n = 46; 11.7%) and 'metastasis' (n = 36; 9.2%) (Figure 1 and Additional file 1).

Next, we identified the large-scale miRNA target networks by uploading targets greater than 100 per individual miRNA onto KeyMolnet (Table 1). Fifty-two miRNAs that construct such a large-scale miRNA target network include let-7, miR-9, 17, 19, 20, 26, 27, 29, 30, 32, 92, 93, 96, 98, 101, 106b, 124, 137, 147, 153, 218, 372, 429, 495, 506, 519, 520, 603, and their closely-related family members. The targets of these miRNAs established highly complex molecular networks, in which the pathways of 'transcriptional regulation by RB/E2F', 'transcriptional regulation by Ets-domain family', and 'transcriptional regulation by p53', the diseases of 'chronic myelogenous leukemia' and 'viral myocarditis', and the pathological event of 'cancer' were notably accumulated (Table 1). Importantly, distinct members belonging to the same miRNA family, for example, five miR-30 family members ranging from miR-30a to miR-30e constructed a virtually identical molecular network (Table 1).

**Table 1:** The large-scale human microRNA target networks

MicroRNA	Number of Targets	Molecules in KeyMolnet Networks	Top Pathway	Score	p-Value	Top Disease	Score	p-Value	Top Pathological Event	Score	p-Value
hsa-let-7a	244	1022	Transcriptional regulation by p53	593	2.69E-179	Viral myocarditis	113	1.21E-34	Cancer	206	1.31E-62

hsa-let-7b	242	1016	Transcriptional regulation by p53	594	1.83E-179	Viral myocarditis	113	9.32E-35	Cancer	206	7.66E-63

hsa-let-7c	243	1020	Transcriptional regulation by p53	593	2.49E-179	Viral myocarditis	113	1.11E-34	Cancer	206	1.10E-62

hsa-let-7d	145	885	Transcriptional regulation by RB/E2F	836	2.18E-252	Chronic myelogenous leukemia	72	1.95E-22	Cancer	130	9.68E-40

hsa-let-7e	236	1111	Transcriptional regulation by p53	575	8.90E-174	Viral myocarditis	116	1.20E-35	Cancer	175	1.86E-53

hsa-let-7f	244	1022	Transcriptional regulation by p53	593	2.69E-179	Viral myocarditis	113	1.21E-34	Cancer	206	1.31E-62

hsa-let-7g	245	1022	Transcriptional regulation by p53	593	2.69E-179	Viral myocarditis	113	1.21E-34	Cancer	206	1.31E-62

hsa-let-7i	245	1022	Transcriptional regulation by p53	593	2.69E-179	Viral myocarditis	113	1.21E-34	Cancer	206	1.31E-62

hsa-miR-9	352	1115	Transcriptional regulation by PPARa	340	5.28E-103	Hepatocellular carcinoma	72	1.69E-22	Cancer	171	3.50E-52

hsa-miR-17	195	961	Transcriptional regulation by RB/E2F	971	3.27E-293	Chronic myelogenous leukemia	92	2.83E-28	Cancer	181	3.58E-55

hsa-miR-19a	226	1094	Transcriptional regulation by RB/E2F	760	2.10E-229	Chronic myelogenous leukemia	113	1.26E-34	Cancer	253	7.04E-77

hsa-miR-19b	225	1094	Transcriptional regulation by RB/E2F	760	2.10E-229	Chronic myelogenous leukemia	113	1.26E-34	Cancer	253	7.04E-77

hsa-miR-20a	165	1038	Transcriptional regulation by RB/E2F	856	1.64E-258	Chronic myelogenous leukemia	87	6.09E-27	Cancer	85	3.33E-26

hsa-miR-20b	198	981	Transcriptional regulation by RB/E2F	962	2.35E-290	Chronic myelogenous leukemia	98	3.39E-30	Cancer	183	6.98E-56

hsa-miR-26a	148	672	Transcriptional regulation by RB/E2F	919	1.76E-277	Chronic myelogenous leukemia	107	6.15E-33	Cancer	181	3.20E-55

hsa-miR-26b	148	672	Transcriptional regulation by RB/E2F	919	1.76E-277	Chronic myelogenous leukemia	107	6.15E-33	Cancer	181	3.20E-55

hsa-miR-27a	229	1192	Transcriptional regulation by CREB	1022	2.23E-308	Chronic myelogenous leukemia	95	1.96E-29	Cancer	194	3.05E-59

hsa-miR-27b	261	1337	Transcriptional regulation by CREB	1022	2.23E-308	Chronic myelogenous leukemia	94	4.51E-29	Cancer	211	4.11E-64

hsa-miR-29a	119	543	Transcriptional regulation by Ets-domain family	430	4.36E-130	Glioma	85	3.46E-26	Cancer	139	1.41E-42

hsa-miR-29b	118	578	Transcriptional regulation by Ets-domain family	422	1.15E-127	Glioma	82	1.55E-25	Cancer	146	1.44E-44

hsa-miR-29c	118	543	Transcriptional regulation by Ets-domain family	430	4.36E-130	Glioma	85	3.46E-26	Cancer	139	1.41E-42

hsa-miR-30a	455	1494	Transcriptional regulation by RB/E2F	777	9.43E-235	Chronic myelogenous leukemia	86	1.11E-26	Cancer	195	2.39E-59

hsa-miR-30b	455	1480	Transcriptional regulation by RB/E2F	781	1.08E-235	Chronic myelogenous leukemia	87	7.01E-27	Cancer	188	1.92E-57

hsa-miR-30c	454	1495	Transcriptional regulation by RB/E2F	778	6.13E-235	Chronic myelogenous leukemia	86	1.15E-26	Cancer	191	3.63E-58

hsa-miR-30d	452	1491	Transcriptional regulation by RB/E2F	778	7.28E-235	Chronic myelogenous leukemia	86	1.01E-26	Cancer	195	1.96E-59

hsa-miR-30e	455	1481	Transcriptional regulation by RB/E2F	780	1.29E-235	Chronic myelogenous leukemia	87	7.25E-27	Cancer	188	2.05E-57

hsa-miR-32	261	905	Transcriptional regulation by RB/E2F	842	2.74E-254	Gastric cancer	80	8.85E-25	Cancer	157	4.19E-48

hsa-miR-92a	219	642	Transcriptional regulation by MEF2	335	1.51E-101	Viral myocarditis	59	1.62E-18	Epithelial-mesenchymal transition	83	7.76E-26

hsa-miR-92b	258	701	Transcriptional regulation by MEF2	328	1.59E-99	Viral myocarditis	60	1.23E-18	Cancer	94	3.97E-29

hsa-miR-93	195	958	Transcriptional regulation by RB/E2F	972	2.37E-293	Chronic myelogenous leukemia	92	2.47E-28	Cancer	181	2.77E-55

hsa-miR-96	142	688	Transcriptional regulation by Ets-domain family	407	3.42E-123	Viral myocarditis	36	1.06E-11	Cancer	106	1.37E-32

hsa-miR-98	162	671	Transcriptional regulation by Myb	549	4.73E-166	Viral myocarditis	85	2.66E-26	Cancer	126	1.42E-38

hsa-miR-101	188	806	Transcriptional regulation by AP-1	492	1.10E-148	Hepatocellular carcinoma	70	6.40E-22	Cancer	127	4.26E-39

hsa-miR-106b	164	1028	Transcriptional regulation by RB/E2F	854	7.21E-258	Chronic myelogenous leukemia	87	5.48E-27	Cancer	85	2.93E-26

hsa-miR-124	285	1346	Transcriptional regulation by RB/E2F	756	3.57E-228	Chronic myelogenous leukemia	83	9.34E-26	Cancer	185	1.90E-56

hsa-miR-137	288	941	Transcriptional regulation by MITF family	339	1.19E-102	Adult T cell lymphoma/leukemia	66	1.30E-20	Cancer	179	1.00E-54

hsa-miR-147	199	867	Transcriptional regulation by RB/E2F	805	4.06E-243	Chronic myelogenous leukemia	113	6.60E-35	Cancer	132	2.57E-40

hsa-miR-153	154	1019	Transcriptional regulation by Myb	507	2.35E-153	Multiple myeloma	60	6.44E-19	Cancer	174	4.31E-53

hsa-miR-218	155	830	Transcriptional regulation by AP-1	344	2.28E-104	Hepatocellular carcinoma	69	1.63E-21	Cancer	136	1.52E-41

hsa-miR-372	101	562	Transcriptional regulation by RB/E2F	1022	2.23E-308	Chronic myelogenous leukemia	85	1.90E-26	Cancer	144	2.75E-44

hsa-miR-429	123	634	Transcriptional regulation by RB/E2F	918	2.45E-277	Chronic myelogenous leukemia	76	1.71E-23	Cancer	130	5.28E-40

hsa-miR-495	156	601	Transcriptional regulation by Ets-domain family	431	2.14E-130	Rheumatoid arthritis	77	5.90E-24	Adipogenesis	79	1.32E-24

hsa-miR-506	394	1536	Transcriptional regulation by Ets-domain family	317	4.69E-96	Viral myocarditis	99	1.73E-30	Cancer	172	1.43E-52

hsa-miR-519a	281	1256	Transcriptional regulation by RB/E2F	811	5.32E-245	Chronic myelogenous leukemia	106	1.34E-32	Cancer	220	8.03E-67

hsa-miR-519b-3p	281	1256	Transcriptional regulation by RB/E2F	811	5.32E-245	Chronic myelogenous leukemia	106	1.34E-32	Cancer	220	8.03E-67

hsa-miR-519c-3p	281	1256	Transcriptional regulation by RB/E2F	811	5.32E-245	Chronic myelogenous leukemia	106	1.34E-32	Cancer	220	8.03E-67

hsa-miR-520a-3p	184	690	Transcriptional regulation by RB/E2F	1022	2.23E-308	Chronic myelogenous leukemia	94	6.95E-29	Cancer	146	1.12E-44

hsa-miR-520b	182	690	Transcriptional regulation by RB/E2F	1022	2.23E-308	Chronic myelogenous leukemia	94	6.95E-29	Cancer	146	1.12E-44

hsa-miR-520c-3p	182	690	Transcriptional regulation by RB/E2F	1022	2.23E-308	Chronic myelogenous leukemia	93	9.28E-29	Cancer	145	1.77E-44

hsa-miR-520d-3p	183	690	Transcriptional regulation by RB/E2F	1022	2.23E-308	Chronic myelogenous leukemia	94	6.95E-29	Cancer	146	1.12E-44

hsa-miR-520e	184	690	Transcriptional regulation by RB/E2F	1022	2.23E-308	Chronic myelogenous leukemia	94	6.95E-29	Cancer	146	1.12E-44

hsa-miR-603	252	1150	Transcriptional regulation by Ets-domain family	344	3.26E-104	Multiple myeloma	84	4.36E-26	Cancer	161	4.24E-49

Among 1,223 human miRNAs examined, Diana-microT 3.0 predicted reliable targets from 273 miRNAs. Among them, KeyMolnet extracted molecular networks from 232 miRNAs. The generated network was compared side by side with human canonical networks of the KeyMolnet library, composed of 430 pathways, 885 diseases, and 208 pathological events. The canonical pathways, diseases, and pathological events with the most statistically significant contribution to the extracted network are shown. The table contains only the large-scale miRNA target networks generated by importing targets greater than 100 per individual miRNA into KeyMolnet. See Additional file 1 for all the information on 232 miRNAs and their target networks.

### Biological Implications of MicroRNA Target Networks

As described above, the present observations indicated that a set of miRNA target genes regulated by an individual miRNA generally constitute the biological network of functionally-associated molecules in human cells. Therefore, it is highly important to obtain deeper insights into biological implications of miRNA target networks.

**Figure 2:**
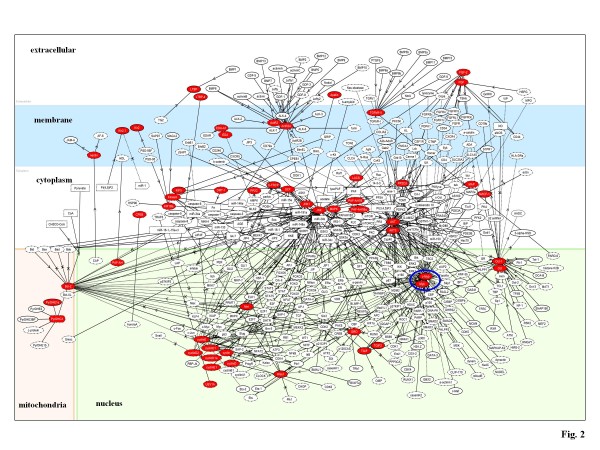
**Molecular network of miR-15a targets**. By the "neighboring" network-search algorithm, KeyMolnet illustrated a highly complex network of miR-15a targets that has the most statistically significant relationship with the pathway of 'transcriptional regulation by myb'. Red nodes represent miR-15a direct target molecules predicted by Diana-microT 3.0, whereas white nodes exhibit additional nodes extracted automatically from the core contents of KeyMolnet to establish molecular connections. The molecular relation is indicated by solid line with arrow (direct binding or activation), solid line with arrow and stop (direct inactivation), solid line without arrow (complex formation), dash line with arrow (transcriptional activation), and dash line with arrow and stop (transcriptional repression). The transcription factor myb is highlighted by a blue circle.

The protooncogene c-myb is a key transcription factor for normal development of hematopoietic cells. A recent study showed that miR-15a targets c-myb, while c-myb binds to the promoter of miR-15a, providing an autoregulatory feedback loop in human hematopoietic cells [17]. Consistent with this study, we found 'transcriptional regulation by myb' as the most relevant pathway to the miR-15a target network (the score = 602; the score p-value = 7.39E-182) (Figure 2 and Additional file 1). These observations propose a scenario that miR-15a synchronously downregulates both c-myb itself and downstream genes transcriptionally regulated by c-myb, resulting in efficient inactivation of the whole molecular network governed by the hub gene c-myb. These results suggest a collaborative regulation of gene expression at both transcriptional and posttranscriptional levels that involve coordinated regulation by miRNAs and transcription factors.

**Figure 3:**
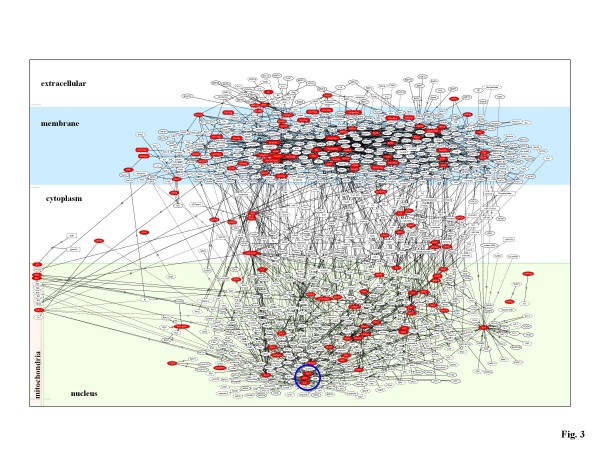
**Molecular network of miR-106b targets**. By the "neighboring" network-search algorithm, KeyMolnet illustrated a highly complex network of miR-106b targets that has the most statistically significant relationship with the pathway of 'transcriptional regulation by Rb/E2F'. Red nodes represent miR-106b direct target molecules predicted by Diana-microT 3.0, whereas white nodes exhibit additional nodes extracted automatically from the core contents of KeyMolnet to establish molecular connections. The molecular relation is indicated by solid line with arrow (direct binding or activation), solid line with arrow and stop (direct inactivation), solid line without arrow (complex formation), dash line with arrow (transcriptional activation), and dash line with arrow and stop (transcriptional repression). The transcription factor E2F is highlighted by a blue circle.

The retinoblastoma protein Rb/E2F pathway acts as a gatekeeper for G1/S transition in the cell cycle. The Rb/E2F-regulated G1 checkpoint control is often disrupted in cancer cells. A recent study showed that miR-106b is directly involved in posttranscriptional regulation of E2F1 [18]. E2F1 activates transcription of miR-106b, while miR-106b targets E2F1, serving as a miRNA-directed negative feedback loop in gastric cancer cells [18]. Supporting these findings, we identified 'transcriptional regulation by Rb/E2F' as the most relevant pathway to the miR-106b target network (the score = 854; the score p-value = 7.21E-258) (Figure 3, Table 1 and Additional file 1). The relationship between miR-106b and Rb/E2F would provide another example of coordinated regulation of gene expression by miRNAs and transcription factors.

We found 'transcriptional regulation by p53' as the most relevant pathway to the target network of all let-7 family members except for let-7d (Table 1). It is worthy to note that the tumor suppressor p53 regulates the expression of components of the miRNA-processing machinery, such as Drosha, DGCR8, Dicer, and TARBP2, all of which have p53-reponsive elements in their promoters [19]. Furthermore, Dicer and TARBP2, along with p53, serve as a target of the let-7 family miRNAs, suggesting a close link between p53 and let-7 in miRNA biogenesis [19]. The expression of let-7 family members was greatly reduced in certain cancer cells [20].

The micropthalmia associated transcription factor (MITF), a basic helix-loop-helix zipper (bHLH-Zip) transcription factor, acts as not only a master regulator of melanocyte differentiation but also an oncogene promoting survival of melanoma. Recent studies indicate that MITF is a direct target of both miR-137 and miR-148b [21,22]. Again, we identified 'transcriptional regulation by MITF family' as the most relevant pathway to both miR-137 (the score = 339; the score p-value = 1.19E-102) and miR-148b (the score = 40; the score p-value = 3.91E-142) target networks (Table 1 and Additional file 1).

Cellular responsiveness to glucocorticoids (GCs) is regulated by the delicate balance of the glucocorticoid receptor (GR) protein, GR coactivators and corepressors, GR splice variants and isoforms, and regulators of GR retrograde transport to the nucleus. A recent study showed that miR-18a targets the GR protein, and thereby inhibits GR-mediated biological events in neuronal cells [23]. Consistent with this, we found 'transcriptional regulation by GR' as the most relevant pathway to the miR-18a target network (the score = 1022; the score p-value = 2.23E-308) (Additional file 1).

Zinc finger transcription factors ZEB1 and ZEB2 act as a transcriptional repressor of E-cadherin. A recent study showed that the expression of miR-200b, which targets both ZEB1 and ZEB2, was downregulated in the cells that undergo TGF-beta-induced epithelial to mesenchymal transition (EMT), and was lost in invasive breast cancer cells [24]. We identified 'transcriptional regulation by ZEB' as the third-rank significant pathway (the score = 155; the score p-value = 1.88E-47) and 'EMT' as the third-rank significant pathological event relevant to the miR-200b target network (the score = 61; the score p-value = 4.15E-19) (Additional file 1).

## Discussion

In general, a single miRNA concurrently downregulates hundreds of target mRNAs by binding to the corresponding 3'UTR of mRNA via either perfect or imperfect sequence complementarity [3]. Such fuzzy mRNA-miRNA interactions result in the redundancy of miRNA-recognized targets. By targeting multiple transcripts and affecting expression of numerous proteins at one time, miRNAs regulate a wide range of cellular functions, such as development, differentiation, proliferation, apoptosis and metabolism. Therefore, we have the question whether a set of miRNA target genes regulated by an individual miRNA generally constitute the biological network of functionally-associated molecules or simply reflect a random set of functionally-independent genes. If the former is the case, what kind of biological networks does the human microRNAome most actively regulates?

To address these questions, first we identified the set of credible target genes for all individual human miRNAs by using the Diana-microT 3.0 program. Then, we investigated miRNA target networks by applying them to KeyMolnet, a bioinformatics tool for analyzing molecular interactions on the comprehensive knowledgebase. Diana-microT 3.0 identified highly reliable targets from 273 miRNAs out of 1,223 all human miRNAs. Previous studies showed that the list of predicted targets for each miRNA varies among different miRNA target prediction programs armed with distinct algorithms, such as TargetScan 5.1 http://www.targetscan.org, PicTar (pictar.mdc-berlin.de), miRanda http://www.microrna.org and Diana-microT 3.0 [25]. Therefore, miRNA target networks are to some extent flexible, depending on the target prediction program employed. Among the programs described above, we have chosen Diana-microT 3.0 because of the highest ratio of correctly predicted targets over other prediction tools and the simplicity of setting a cut-off point for detection of reliable miRNA-target interactions based on the miTG score [11].

Here we found that highly reliable targets of substantial numbers of human miRNAs actually constructed biologically meaningful molecular networks. These observations strongly supported the theoretical view that miRNA target genes regulated by an individual miRNA in the whole human microRNAome generally constitute the biological network of functionally-associated molecules. A recent study showed that interacting proteins in the human PPI network tend to share restricted miRNA target-site types than random pairs, being consistent with our observations [26].

We also found that there exists a coordinated regulation of gene expression at the transcriptional level by transcription factors and at the posttranscriptional level by miRNAs in miRNA target networks. Recently, Cui et al. investigated the relationship between miRNA and transcription factors in gene regulation [27]. Importantly, they found that the genes with more transcription factor-binding sites have a higher probability of being targeted by miRNAs and have more miRNA-binding sites.

A recent study by miRNA expression profiling of thousands of human tissue samples revealed that diverse miRNAs constitute a complex network composed of coordinately regulated miRNA subnetworks in both normal and cancer tissues, and they are often disorganized in solid tumors and leukemias [28]. During carcinogenesis, various miRNAs play a central role, acting as either oncogenes named oncomir or tumor suppressors termed anti-oncomir, by targeting key molecules involved in apoptosis, cell cycle, cell adhesion and migration, chromosome stability, and DNA repair [5]. Many miRNA gene loci are clustered in cancer-associated genomic regions [29]. Furthermore, miRNA expression signatures well discriminate different types of cancers with distinct clinical prognoses [30]. In the present study, KeyMolnet analysis of miRNA target networks showed that the most relevant pathological event is 'cancer', when top three pathological events were overall cumulated. Furthermore, the highly relevant diseases include 'adult T cell lymphoma/leukemia', 'chronic myelogenous leukemia', and 'hepatocellular carcinoma'. These observations suggest that the human microRNAome plays a more specialized role in regulation of oncogenesis. Therefore, the miRNA-based therapy directed to targeting multiple cancer-associated pathways simultaneously might serve as the most effective approach to suppressing the oncogenic potential of a wide range of cancers.

## Conclusion

The reliable targets predicted by Diana microT 3.0 derived from approximately 20% of all human miRNAs constructed biologically meaningful molecular networks by KeyMolnet. These observations support the view that miRNA target genes regulated by an individual miRNA in the whole human microRNAome generally constitute the biological network of functionally-associated molecules. In the human miRNA target networks, the most relevant pathway is transcriptional regulation by transcription factors RB/E2F, the disease is adult T cell lymphoma/leukemia, and the pathological event is cancer. In miRNA target networks, there exists a coordinated regulation of gene expression at the transcriptional level by transcription factors and at the posttranscriptional level by miRNAs.

## Competing interests

The authors declare that they have no competing interests.

## Authors' contributions

JS designed the methods, analyzed the data, and drafted the manuscript. HT helped the data analysis. All authors have read and approved the final manuscript.

## Supplementary Material

Additional file 1 **KeyMolnet identifies microRNA target networks in 232 human miRNAs**. The prediction of target genes of individual miRNA was performed by Diana-microT 3.0. Entrez Gene IDs of miRNA target genes were uploaded onto KeyMolnet. The generated network was compared side by side with human canonical networks composed of 430 pathways, 885 diseases, and 208 pathological events of the KeyMolnet library. Top-three pathways, diseases, and pathological events with the statistically significant contribution to the extracted network are shown. Click here for file
